# Autologous haematopoietic stem cell transplantation for multiple sclerosis in the UK: A 20‐year retrospective analysis of activity and haematological outcomes from the British Society of Blood and Marrow Transplantation and Cellular Therapy (BSBMTCT)

**DOI:** 10.1111/bjh.20199

**Published:** 2025-06-11

**Authors:** Majid Kazmi, Paolo A. Muraro, Varun Mehra, Ian Gabriel, Eleonora De Matteis, Gavin Brittain, Alice Mariottini, Richard Nicholas, Eli Silber, Julia Lee, Rachel Pearce, Ruth Paul, Maria Pia Sormani, Alessio Signori, Victoria Potter, Eduardo Olavarria, Ram Malladi, Basil Sharrack, John A. Snowden

**Affiliations:** ^1^ Guy's & St Thomas' NHS Trust London UK; ^2^ Department of Brain Sciences Imperial College London London UK; ^3^ Imperial College Healthcare NHS Trust London UK; ^4^ King's College Hospital NHS Trust London UK; ^5^ Sheffield Teaching Hospitals NHS Foundation Trust Sheffield UK; ^6^ Sheffield Institute for Translational Neuroscience, University of Sheffield Sheffield UK; ^7^ Department of Neurosciences, Psychology, Drug Research and Child Health (NEUROFARBA) University of Florence Florence Italy; ^8^ British Society of Blood and Marrow Transplantation and Cellular Therapy London UK; ^9^ University of Genova Genoa Italy; ^10^ IRCCS Ospedale Policlinico San Martino Genoa Italy; ^11^ Cambridge University Hospitals NHS Trust Cambridge UK; ^12^ Department of Haematology Sheffield Teaching Hospitals NHS Foundation Trust Sheffield UK

**Keywords:** autoimmune disease, haematopoietic stem cell transplantation, multiple sclerosis, stem cells

## Abstract

Autologous haematopoietic stem cell transplantation (AHSCT) has been developed as a treatment for multiple sclerosis (MS) since 1995. The United Kingdom is one of the most active countries performing AHSCT for MS in Europe. We report the UK experience of AHSCT for MS in 364 patients with MS treated with AHSCT between 2002 and 2023. We report transplant‐related mortality (TRM), AHSCT complications and efficacy as defined by expanded disability status scale (EDSS) progression‐free survival (PFS) at 2 years and 5 years. 209 (58%) had relapsing–remitting MS (RRMS) and 130 (36%) had progressive MS. Median EDSS at the time of HSCT was 6.0 (range: 0–9) and duration of disease was 10 years (range: 4–34). TRM was 1.4%, exclusively occurred in patients with advanced baseline disability (median EDSS: 6.5). Epstein–Barr virus (EBV) reactivation occurred in 75.9% of patients where EBV results were reported (235/311). Overall PFS was 83.5% at 2 years post‐HSCT and 62.4% at 5 years. This large study demonstrates the evolution of this one‐off treatment across the United Kingdom, its safety and sustained efficacy in patients with severe/refractory MS. The uneven geographical access is a future consideration in equitable delivery across the UK NHS as the evidence base for AHSCT in MS treatment pathways becomes stronger.

## Graphical abstract

We report the UK experience of autologous haematopoietic stem cell transplantation (AHSCT) for multiple sclerosis (MS) in 364 patients. In our observational study, transplant‐related mortality was 1.4%, Epstein–Barr virus reactivation occurred in 75.9%, while disability progression‐free survival was 83.5% at 2 years post‐AHSCT and 62.4% at 5 years. We demonstrate the evolution of this one‐off treatment across the United Kingdom, its safety and sustained efficacy in patients with severe/refractory MS.

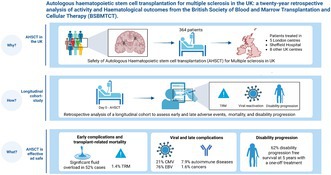

## INTRODUCTION

Multiple sclerosis (MS) is a chronic disease of the central nervous system (CNS) and the main cause of disability in the working‐age population.^1^ MS leads to a 7‐year reduction in life expectancy and a threefold increase in all‐cause mortality.^2^ MS prevalence in the United Kingdom exceeds 150 000, with a global incidence of 3.6 cases per 100 000 person‐years.^3^, ^4^

MS presents as relapsing‐remitting MS (RRMS) in approximately 85% of cases, beginning in the third decade with a female to male ratio of 2.3:1,^4^ or as primary progressive MS (PPMS) with typical onset in the fourth decade.^5^, ^6^, ^7^ RRMS involves episodes of neurological symptoms (relapses) followed by recovery. PPMS is marked by accumulation of disability without distinct relapses. Over time, many RRMS patients transition to secondary progressive MS (SPMS), with steady progression of disability.

Evidence exists for autoimmune pathogenesis, with both T and B lymphocytes implicated through activation, cytokine production, CNS trafficking and consequent neuroinflammation.^8^, ^9^ Associations with EBV infection support a potential trigger for the aberrant immunity.^10^ Early effective therapy is key to countering neurodegenerative processes and progressive disability that characterises SPMS. High‐efficacy disease‐modifying therapies (DMTs) for RRMS, including alemtuzumab (anti‐CD52),^11^ ocrelizumab (anti‐CD20),^12^ ofatumumab (anti‐CD20)^13^ and natalizumab (anti‐ α4‐integrin),^14^ reduce relapse frequency and associated disability and can be used first line in some patients.^15^ They are not curative, require long‐term use, pose cumulative risks, high healthcare costs and ultimately disease progression independent of relapse activity (PIRA) occurs.^16^, ^17^

Autologous haematopoietic stem cell transplantation (AHSCT) is a one‐off therapy that achieves sustained disease remission in patients with MS (pwMS).^18^, ^19^ AHSCT enables immune reset by ablating pathogenic immune cells with reconstitution of a self‐tolerant immune system.^18^, ^19^ The extent of immune cell ablation depends on conditioning regimen intensity, which targets the lymphoid compartment alone or both lymphoid and myeloid compartments.^18^ Moderate‐ and low‐intensity conditioning regimens are mostly used in the treatment of MS due to favourable tolerability and efficacy profiles.^20^

AHSCT has proven superior to DMTs in achieving no evidence of disease activity (NEDA) (i.e. absence of relapses, MRI activity and disability progression).^21^, ^22^, ^23^ Recent data from real‐world studies showed 40% of patients maintain NEDA 10 years post‐therapy.^24^ Since AHSCT was first used for MS in the 1990s, the tolerability of the procedure has improved and mortality rates reduced from 7.3% to 0.2%.^20^ However, safety concerns and lack of awareness still restrict AHSCT access.

AHSCT has been commissioned through the NHS for severe MS since 2013^25^; despite this, uptake in the UK remains low, with less than 0.3% of patients potentially eligible undergoing the procedure up to 2024. This is an underestimate as a percentage of patients travelled abroad to receive AHSCT due to a lack of perceived access.^26^ The Mexico group published on 1700 patients having AHSCT for MS, and 18.8% were from the United Kingdom (319 patients) and similar numbers are likely to have travelled to Russia and other sites.^26^ Since 2016, there has been a significant upturn in AHSCT activity for MS, driven by raised awareness, commissioning guidance and publication of several papers.^22^, ^23^, ^27^, ^28^

We report the UK‐wide experience of AHSCT for MS from 2002 to 2023, highlighting outcomes, toxicities, access factors and identifying factors associated with better tolerability and efficacy that can help improve clinical practice.

## METHODS AND STATISTICAL ANALYSIS

### Patient selection

We retrospectively collected data from patients treated with AHSCT at 14 participating centres between 2002 and 2023. Patients' eligibility for AHSCT was adjudicated on a case‐by‐case basis by a multidisciplinary team (MDT), including neurologists and haematologists, according to recognised principles^18^ based on active disease, relapses or MRI activity, defined by new T2 and/or gadolinium enhancing lesions, despite DMT, or ‘aggressive’ disease if treatment naive, and fit for AHSCT. All patients signed informed consent to treatment and data collection in accordance with the Declaration of Helsinki. Patients on active clinical trials were not included.

All UK transplant centres reporting autologous AHSCT for MS activity to the BSBMTCT/EBMT databases during the study period were invited to participate. Supporting Information data were collected with an encrypted password‐protected Excel spreadsheet sent to each participating centre. Fully anonymised data returned from the centres were checked for consistency by the study team.

### Treatment procedure

Stem cell mobilisation was predominantly cyclophosphamide (dose range: 2–4 g/m^2^) followed by G‐CSF daily at a dose of 5–10 μm/kg daily starting 24 h post‐cyclophosphamide for 7–10 days. G‐CSF only mobilisation (10 μm/kg) was allowed for those failing cyclophosphamide‐based mobilisation.

Patients went through standard pretransplant work‐up before admission for AHSCT. Conditioning was cyclophosphamide/ATG with cyclophosphamide 200 mg/kg based on ideal body weight and rabbit‐ATG (r‐ATG, Thymoglobulin, Sanofi) at either 6.0 mg/kg or 7.5 mg/kg or carmustine/etoposide/cytarabine/melphalan regimen plus an equivalent dose of rATG (BEAM‐ATG). Stem cell reinfusion was delivered with a minimum dose of 2.0 × 10^6^/kg CD34+ cells following a 24‐h wash‐out. Supportive care and monitoring (including platelet and packed red cell transfusions, antimicrobial prophylaxis, management of fever, dietetics and physiotherapy support) were provided as per centre protocols. All centres were accredited by JACIE (Joint Accreditation Committee of the International Society for Cellular Therapy (ISCT) and European society for Blood and Marrow Transplantation (EBMT)).

### Statistical analysis

Data were analysed in Stata 18 (StataCorp. 2023. *Stata Statistical Software*: *Release 18*. College Station, TX: StataCorp LLC.) Survival and other time‐to‐events (e.g. time to EDSS progression) were calculated by Kaplan–Meier, and comparisons between groups were made by Cox regression. EDSS progression was calculated from serial EDSS assessments, and patients were deemed to be at risk for progression from +90 days post‐transplant. Interaction terms were used to assess the effects of viral reactivation (which took place before 90 days) and ATG dose on EDSS progression. Comparisons between viral reactivation rates were made by logistic regression. Results were reported as hazard ratio (HR) together with the 95% confidence interval (CI).

### Outcomes

This analysis focuses on the safety and transplant‐related complications of AHSCT, with efficacy data restricted to progression‐free survival described below. More detailed analysis of neurological outcomes is the focus of another manuscript under consideration elsewhere.^29^

### Efficacy

Clinical effectiveness was assessed as progression‐free survival (PFS) in the entire cohort and by MS phenotype. Progression‐free survival was defined as the absence of disability progression—an increase in EDSS score by 0.5 points if the baseline extended disability status scale (EDSS) score was ≥6.0 and by 1 point if the baseline EDSS score was <6.0, confirmed 6 months after. Each event was adjudicated by a local neurologist based on examination and medical records review.

### Safety

Significant adverse events during the in‐patient stay, first 100 days post AHSCT, and then, late effects beyond day 100 were all recorded. Specifically, we collected data on the incidence of fever >38.0°C occurring anytime during the AHSCT procedure; rATG reactions and significant fluid overload (defined by >5% weight gain +/− peripheral or central oedema and need for diuresis); moderate to severe (Grade ≥ 2) nausea and diarrhoea. Data on viral reactivations assessed by whole blood PCR monitoring for EBV and CMV DNA were also collected. Clinically significant CMV viraemia was defined as CMV DNA copies >1000/mL or >3 Log copies/mL on two consecutive readings and/or where antiviral treatment was required. EBV viral load >300 000 copies/mL (or 30 000 IU/mL using WHO standard PCR) or where treatment with anti‐CD20 therapy (rituximab) was required for EBV‐related symptoms was considered clinically significant for the purpose of this study.

Transplant‐related mortality (TRM) was defined as all deaths within 100 days of stem cell re‐infusion to include deaths occurring after commencing conditioning but prior to stem cell reinfusion. Specific late effect information was collected on the incidence of a secondary autoimmune disease and any new cancer diagnosis at any stage post AHSCT.

## RESULTS

Three hundred and sixty‐four patients with MS (pwMS) were included in this analysis from 14 transplant centres. 210 (58%) were female, median age at transplant was 40 years (range: 18–66, IQR: 33–47). 209 (58%) had RRMS, 130 (36%) had progressive MS (47 PPMS; 83 SPMS) and 25 patients' subtype was not recorded (6%). Median EDSS at time of AHSCT was 6.0 (range: 0–9) and disease duration of 10 years (range: 4–34 years) (Table 1).

**TABLE 1:** Demographics and transplant.

Factor	Level	Patients		Type of MS					
	Unless o/w stated	*N* (unless o/w stated)	% (unless o/w stated)	Secondary progressive		Primary progressive		Relapsing remitting	
Total		364		83		47		209	
Patient sex	Male	154	42%	38	46%	34	72%	70	33%
	Female	210	58%	45	54%	13	28%	139	67%
Age at transplant		Median	Range	Median	Range	Median	Range	Median	Range
		40 year	19–66 year	43 year	25–62 year	47 year	31–64 year	38 year	19–66 year
		Mean/SD	IQR	Mean/SD	IQR	Mean/SD	IQR	Mean/SD	IQR
		40 year 9.4 year	33–47 year	44 year 7.9 year	38–49 year	46 year 8 year	39–50 year	38 year 9.4 year	31–45 year
By decade	<30	53	15%	5	6%	0		42	20%
	30–39	123	34%	21	25%	12	26%	76	36%
	40–49	127	35%	38	46%	22	47%	63	30%
	50–59	56	15%	18	22%	11	23%	27	13%
	60+	5	1%	1	1%	2	4%	1	0.5%
Length of MS at SCTAHSCT (since 1st symptoms)		Median	Range	Median	Range	Median	Range	Median	Range
		10 year	4 month to 34 year	12 year	3–29 year	7 year	1–31 year	9 year	4 month to 34 year
		Mean/SD	IQR	Mean/SD	IQR	Mean/SD	IQR	Mean/SD	IQR
		10 year/6 year	6–14 year	13 year/5 year	9–17 year	8 year/5 year	4–11 year	10 year/6 year	5–13 year
Age at symptom onset		Median	Range	Median	Range	Median	Range	Median	Range
		29 year	9–56 year	30	9–51	37	19–55	27	9–56
		Mean/SD	IQR	Mean/SD	IQR	Mean/SD	IQR	Mean/SD	IQR
		30 year/9 year	23–36	30/8	25–35	37/8	32–42	28/9	21–34
Year of transplant	2002–2012	13	4%	5	6%	0		8	4%
	2013–2015	29	8%	8	10%	0		21	10%
	2016–2023	322	88%	70	84%	47	100%	180	86%
Baseline EDSS	Median range Mean	Median	Range	Median	Range	Median	Range	Median	Range
		6	0–9	6.125	0–8	6	2–7	5.5	1.5–9
		Mean/SD	IQR	Mean/SD	IQR	Mean/SD	IQR	Mean/SD	IQR
		5.2/1.6	4–6.5	6.0/1.0	6–6.5	5.5/1.3	4.5–6.5	4.9/1.7	3.5–6
	0–4.5	100	31%	5	6%	14	30%	81	41%
	>4.5	223	69%	73	94%	33	70%	117	59%
	Not reported	41		5		0		11	
Different grouping	0–3.0	46	14%	1	1%	3	6%	42	21%
	3.5–5.5	92	28%	13	17%	15	32%	64	32%
	>5.5	185	57%	64	82%	29	62%	92	46%
Prior treatment	Naïve to prior DMT	32	11%	5	7%	17	68%	10	5%
	Unknown prior DMT	70		8		22		15	
Conditioning	Cyclo/ATG/Methyl pred	227	62%	71	89%	41	87%	115	55%
	Cyclo/ATG	125	34%	6	8%	6	13%	88	42%
	BEAM/ATG	9	3%	3	4%	0		6	3%
	*Not reported*	3		3		0		0	
Dose of ATG	≤6 mg/kg	141	39%	23	28%	8	17%	96	46%
	>6 mg/kg	219	61%	58	72%	39	83%	111	54%
	No ATG/not reported	4		2		0		2	

Abbreviations: AHSCT, autologous haematopoietic stem cell transplantation; ATG, anti‐thymocyte globulin; BEAM, bis‐chloro‐ethyl‐nitrosourea (BCNU), cytosine arabinoside, etoposide, melphalan; Cyclo—cyclophosphamide; DMT, disease modifying therapy; EDSS, Expanded Disability Status Scale; IQR, interquartile range; MS, multiple sclerosis; *N*, number; SD, standard deviation.

**FIGURE 1:**
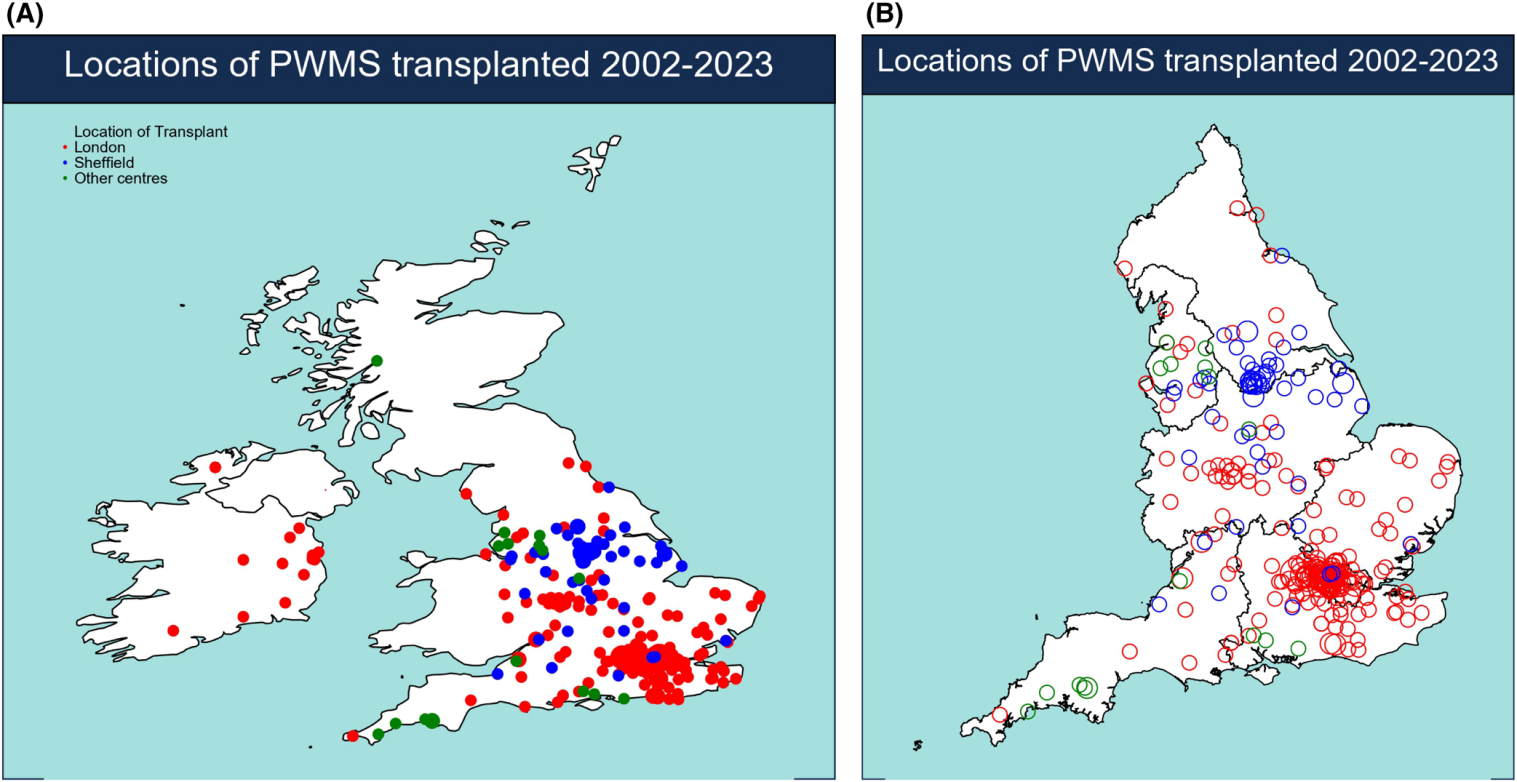
(A) Map showing approximate locations of home addresses of patients receiving autologous stem cell transplantation in the (A) United Kingdom (B) England only.

Figure 1A,B shows geographical location of the most active centres and the patients undergoing AHSCT. Activity was centred around London and Sheffield, which historically have been the most active UK centres for AHSCT in autoimmune diseases. Many patients travelled a significant distance to undergo AHSCT, including 15 from the Republic of Ireland (Figure 1). Assuming uniform incidence of MS across England and Wales (regional incidence data are not available), and using postcode origin of patients, we derived incidence rate of AHSCT/million population (Table 2). This confirmed inequity of access with some regions undertaking markedly less activity compared to the regions around London and Sheffield.

**TABLE 2:** Regions of addresses of PWMS and centres where they were treated.

Regional team/country of home address	Transplant centre: regional team									Region population (2021/2022 Census)	Transplants per 1 000 000 people
	London	Midlands	NE & Yorks	NW	SE	SW	Scotland	Private	Total		
London	83	0	2	0	0	0	0	0	85	8 799 728	9.66
Midlands	27	1	17	0	0	0	0	0	45	10 830 811	4.15
NE & Yorkshire	7	0	31	0	0	0	0	0	38	8 441 200	4.50
NW	7	0	5	6	0	0	0	0	18	7 103 985	2.53
SE	72	0	1	0	3	0	0	0	76	8 977 685	8.47
SW	12	0	4	0	0	7	0	0	23	5 707 515	4.03
East	24	0	2	0	0	0	0	0	26	6 629 125	3.92
England total	232	1	62	6	3	7	0	0	311	56 490 049	5.51
Scotland	0	0	0	0	0	0	1	0	1	5 436 600	0.18
Wales	0	0	0	0	0	0	0	0	0	3 107 500	0
Northern Ireland	0	0	0	0	0	0	0	0	0	1 903 175	0
UK NHS total	232	1	62	6	3	7	1	0	312	66 937 324	4.66
Republic of Ireland	15	0	0	0	0	0	0	0	15	5 149 139^a^	2.91
Unknown^b^	7	1	5	1	0	0	0	22	36	‐	‐
Total UK + Ireland	254	2	67	7	3	7	1	22	363	72 086 463	5.04

Abbreviations: NE, North East; NHS, National Health Service; SE, South East; SW, South West; UK, United Kingdom.

^a^ 2022 census.

^b^ Most of the ‘unknowns’ will likely be private patients.

98% (352/361) of patients received a cyclophosphamide/rATG conditioned AHSCT and 2% (9/361) received BEAM/ATG. 61% (219/360) of patients received an rATG dose of 7.5 mg/kg or more (Table 1). All patients engrafted post stem cell reinfusion. Median time to neutrophil engraftment was 11 days (range: 10–13 days).

### Safety outcomes

Early complications occurred in 97% (253/261), where data were available. Most reported were: fluid overload (>2% body weight gain) in almost all patients (218/221; 99%), clinically significant fluid retention/weight gain in 161/307 patients (52%) and high‐grade fever during conditioning in 86% (225/261 patients) (Table 3).

**TABLE 3:** Transplant outcomes.

Outcome	Level	Patients	% (unless o/w stated)	Type of MS					
	Unless o/w stated	*N*		Secondary progressive		Primary progressive		Relapsing remitting	
Total		364		83		47		209	
		Median	Range	Median	Range	Median	Range	Median	Range
Follow up		3 year 9 month	1 month to 11 year 9 month	4 year 3 month	1 month to 11 year 9 month	4 year 1 month	1 month to 7 year 1 month	3 year 7 month	1 month to 11 year 9 month
Status at follow up	Alive	357		83		45		202	
	Dead (TRM)	4		0		1		3	
	Dead (MS)	2		0		0		2	
	Late death (non‐MS)	1		0		1		0	
Neutrophil engraftment	Yes	361	99%	83	100%	46	100%	207	99%
	No	3^a^	1%			1^a^		2^a^	1%
	Median days	11		11		11.5		11	
	IQR	10–12		10–13		10–13		10–12	
Overall survival	100D	99%	97–100%	100%	‐	96%	84–99%	99%	95–100%
	1 year	99%	97–100%	100%	‐	96%	84–99%	99%	95–100%
	5 year	98%	95%–99%	100%	‐	96%	84–99%	97%	92–99%
	10 year	94%	87–97%	100%	‐	‐	‐	91%	76–97%
OS 5 year by baseline EDSS	0–4.5	100%	‐	100%	‐	100%	‐	100%	‐
	>4.5	96%	91–99%	100%	‐	94%	78–98%	95%	85–98%
OS by continuous EDSS	*p*‐value	*p* = 0.032	HR = 1.86	‐	‐	*p* = 0.320	HR = 5.31	*p* = 0.038	HR = 1.73
Evaluable for EDSS progression	*N* = 271	*N* = 271	*N* = 64		*N* = 39		*N* = 168		
EDSS progression free survival	1 year	91%	86–94%	87%	75–93%	81%	64–91%	94%	89–97%
	2 year	83%	78–86%	77%	63–86%	78%	61–88%	87%	80–92%
	3 year	74%	68–80%	68%	53–79%	61%	42–76%	80%	72–86%
	4 year	68%	61–74%	63%	47–75%	46%	26–64%	75%	66–82%
	5 year	62%	55–69%	59%	43–72%	46%	26–64%	68%	57–76%
	*p*‐value and HR for difference v. RR		*p* = 0.047	HR = 1.69	*p* = 0.014	HR = 2.07		Reference	
Evaluable for EDSS progression 2013–2023	*N* = 262	*N* = 262	*N* = 60		*N* = 39		*N* = 163		
EDSS progression free survival (AHSCT between 2013 and 2023)	1 year	91%	87–94%	88%	76–94%	81%	64–91%	95%	90–97%
	2 year	84%	79–88%	79%	65–88%	78%	61–88%	87%	81–92%
	3 year	74%	68–80%	69%	54–80%	61%	42–76%	80%	72–86%
	4 year	68%	61–75%	63%	47–76%	46%	26–64%	76%	67–82%
	5 year	64%	56–71%	59%	41–73%	46%	26–64%	70%	60–78%
	*p* value for difference v. RR		*p* = 0.039	HR = 1.78	*p* = 0.010	HR = 2.17		Reference	
Transplant related mortality	100D	1%	1–3%	0%		4%	1–16%	2%	0–5%
	1 year	1%	0–3%	0%		4%	1–16%	2%	0–5%
Any EBV reactivation	No	72	23%	20	27%	10	23%	42	22%
	Yes	235	76%	53	72%	33	75%	149	77%
	Primary infection	4	1%	0		1	2%	3	2%
	Not available	53	‐		‐		‐		‐
EBV by ATG dose	ATG dose ≤ 6.0	33/112	29%	8/18	44%	4/8	50%	21/86	24%
	ATG dose ≥ 7.5	39/199	20%	12/55	22%	6/36	17%	21/108	19%
	*p*‐value	0.051		0.075		0.064		0.483	
Any CMV reactivation	No	241	78%	63	89%	26	60%	152	79%
	Yes	66	21%	8	11%	17	40%	41	21%
	Not available	57	‐		‐		‐		‐
CMV by ATG dose	ATG dose ≤ 6.0	9/110	8%	0/18	0%	1/7	14%	8/85	9%
	ATG dose ≥ 7.5	57/197	29%	8/53	17%	16/36	44%	33/108	31%
	*p*‐value	*p*‐0.0005		*p*‐0.100		*p*‐0.215		*p*‐0.0005	
Complications (where data available)									
Fever during priming	Yes	50	20%	16	21%	7	16%	27	21%
	No	199	80%	60	79%	37	84%	102	79%
	Unknown	12		0		1		11	
Fever during conditioning	Yes	225	86%	65	85%	37	80%	123	88%
	No	36	14%	11	14%	9	20%	16	12%
	Unknown	0		0		0		0	
Fever post conditioning	Yes	188	73%	58	77%	31	72%	99	71%
	No	70	27%	17	23%	12	28%	41	29%
	unknown	3		1		1		1	
Grade 2+ Nausea/vomiting	Yes	134	52%	35/75	47%	21/45	47%	78/138	57%
	No	124	48%						
	Unknown	3							
Grade 2+ diarrhoea	Infective	14	5%	3	4%	4	9%	7	5%
	Not infective	81	63%	51	68%	31	69%	81	59%
	None	163	31%	21	28%	10	22%	50	36%
Fluid overload/weight gain	No	146	48%	24	33%	15	37%	107	55%
	Yes	161	52%	48	67%	26	63%	87	45%

Abbreviations: AHSCT, autologous haematopoietic stem cell transplant; ATG, anti‐thymocyte globulin; CMV, cytomegalovirus; EBV, Epstein–Barr virus; EDSS, Expanded Disability Status Scale; HR, hazard ratio; MS, multiple sclerosis; *N*, number; OS, overall survival.

^a^ Three patients died before neutrophil recovery.

There were 5/364 deaths (1.4%) within 100 days of stem cell reinfusion predominantly due to toxicity of the conditioning regimen with acute decompensation leading to cardio‐respiratory failure or dysrhythmia, with two patients dying pre‐stem cell reinfusion. Two of the subjects had PPMS and three reported having RRMS. The median EDSS was 6.5 for this group and the median disease duration was 8 years from diagnosis (range: 5–17 years) and 9 years from first symptoms (range: 6–31 years). The three patients reported as RRMS had a median EDSS score of 6.5 with a median disease duration from first symptoms of 9 years (range: 7–18 years) suggesting they were more likely established secondary progressive MS or transitioning at the time of AHSCT (Table 4).

**TABLE 4:** Mortality.

Age at transplant (years)	43	58	41	51	30
Gender	M	F	F	F	F
Significant medical history	Mild emphysema	Hypothyroidism, hypertension	Nil	Asthma (fatty liver, hypercholesterolaemia)	Asthma
Disease type	PPMS	PPMS	RRMS	RRMS	RRMS
Disease duration (years from diagnosis)	5	17	9	8	5
Disease duration (years from onset of symptoms)	6	31	9	18	7
DMT pre transplant	None	None	Natalizumab, interferon, dimethylfumarate	Copaxone and natalizumab	Alemtuzumab and interferon
EDSS prior to transplant	6.5	6.5	6.5	6.5	6.0
ATG dose (mg/kg) planned/received	7.5/7.5	7.5/5	7.5/7.5	6/6	7.5/7.5
Fluid overload with clinical signs	Yes	Yes	Yes	Yes	Yes
Timing of death	Conditioning	23 days after AHSCT	4 days after AHSCT	Conditioning	54 days after AHSCT
Cause of death	Cardiac arrest and pulmonary oedema	ARDS Chest infection/sepsis	Cardiac arrest, post‐traumatic subarachnoid haemorrhage	Cardiac arrest Dyselectrolytaemia	Sepsis, PLTD

Abbreviations: AHSCT, autologous haematopoietic stem cell transplantation; ARDS, acute respiratory distress syndrome; ATG, anti‐thymocyte globulin; DMT, disease‐modifying therapy; EDSS, Expanded Disability Status Scale; F, female; M, male; PTLD, post‐transplant lymphoproliferative disorder.

### Late deaths beyond day +100

Three further deaths occurred beyond 1 year after transplant due to MS progression in one, and one died of COVID beyond 1 year post AHSCT. The third patient was lost to follow up, with the cause of death unknown. EDSS at the time of AHSCT was 6, 6.5 and 7.5, reflecting significantly advanced disease.

### Late effects

Following AHSCT, 5/315 (1.6%) patients were diagnosed with new malignancies; skin cancers (*n* = 2 at 14 months post‐transplant and at unknown date), T‐acute lymphoblastic leukaemia (T‐ALL) (*n* = 1 at 4 years post‐transplant), prostate cancer (*n* = 1 at 5 years post‐transplant) and breast cancer (*n* = 1 at 2 years post‐transplant). 24/305 (7.9%) patients developed secondary autoimmune disease predominantly thyroid disease (*n* = 21), immune thrombocytopenia (*n* = 2) and coeliac disease (*n* = 1).

### Viral reactivations

CMV reactivation (>10 copies/mL DNA) was detected in 66/307 cases (21%) and data were not available in 56 cases (15%). Clinically significant CMV reactivation (defined above) occurred in 47/66 cases (15% of patients where we had data), but no CMV disease was observed. CMV reactivation was more commonly associated with a higher rATG dose >6.0 mg/kg (29% vs. 8%. *p* = 0.0005; Table 3).

EBV serological status prior to AHSCT was positive in all cases apart from one indeterminate and one seronegative patient. EBV reactivation (defined by viraemia >10 DNA copies/mL consecutively), as previously described^30^ was demonstrated in 76% of the total group, although four cases (1.1%) were incorrectly described as primary infection (despite serological evidence of EBV pre‐AHSCT). In 53 cases (14.5%), EBV monitoring was not performed. Rates of EBV reactivation were not significantly increased with rATG doses >6.0 mg/kg (24% vs. 71%. *p* = 0.051; Table 3).

Very high levels of EBV reactivation are associated with adverse outcomes.^30^ Of the 307 patients for whom we had EBV monitoring data, 235 EBV reactivation cases occurred with 15 (6%) requiring treatment with rituximab. Rituximab was not used in patients treated in Sheffield and other centres outside London, where a lower total rATG dose of 6.0 mg/kg was deployed, no adverse sequelae of EBV reactivation were reported at these sites. Following adoption of pre‐emptive rituximab after 2019 by the Pan‐London group at a threshold of 500 000 copies/mL (50 000 IU/mL), no cases of clinically significant EBV disease were seen.

### Efficacy outcomes

**FIGURE 2:**
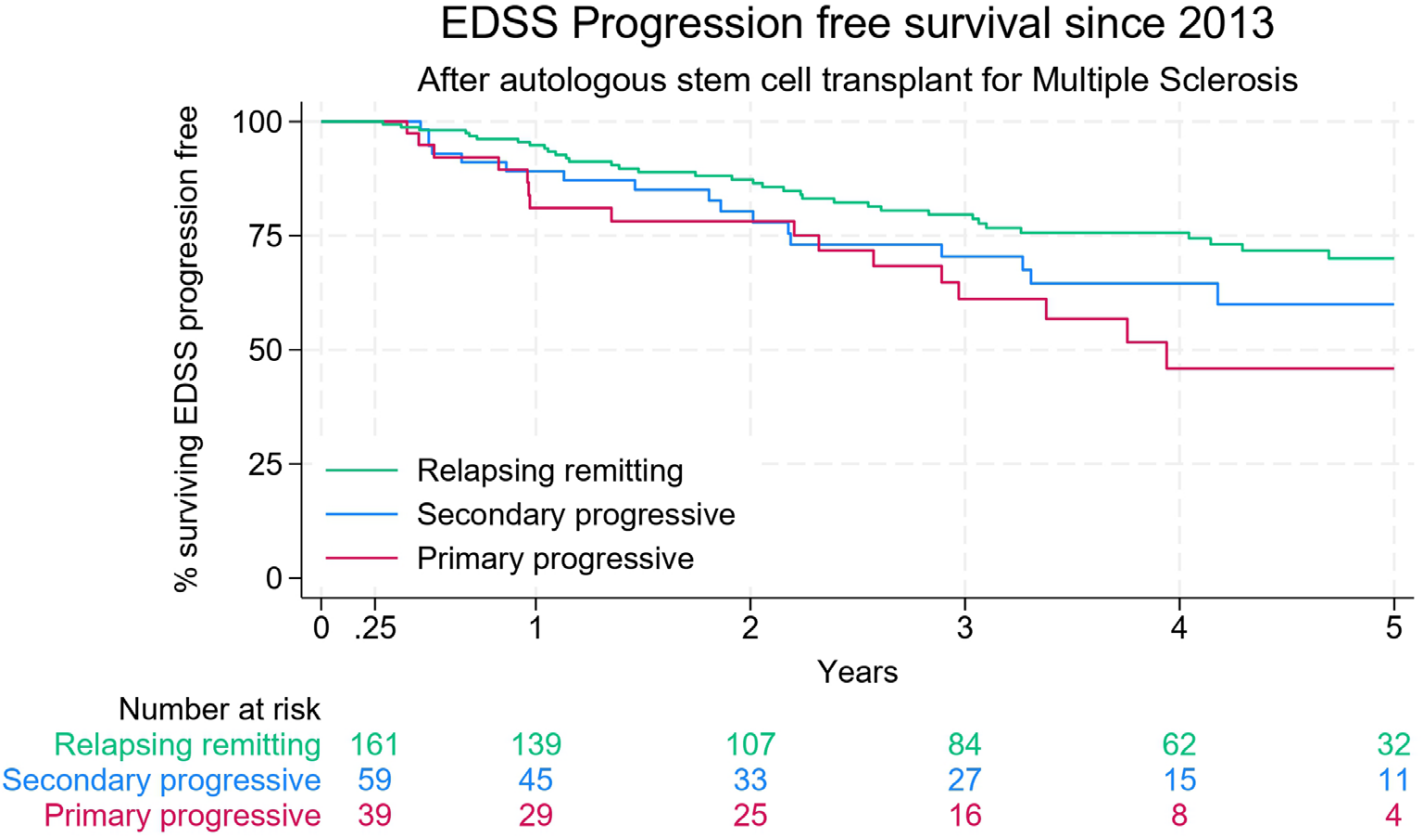
Kaplan–Meier curves of progression‐free survival by subtype. [Colour figure can be viewed at wileyonlinelibrary.com]

Progression‐free survival was 62% (95% CI: 55%–69%) for the overall group at 5 years and significantly higher in RRMS patients compared with PPMS (HR 2.07) and SPMS (HR 1.69) patients (*p*‐0.04). Similar outcomes were noted in patients transplanted pre‐ and post‐2013 (Table 3). Even in the PPMS group, 46% had no EDSS progression 5 years post AHSCT (Table 2, Figure 2). The PFS differences beyond 90 days AHSCT were strikingly better in patients with ATG dose ≤6.0 mg/kg versus higher doses (HR = 2.52, *p*‐0.0005; Figure S1), mainly noted in RRMS patient groups (Table 5). The PFS was also significantly lower in patients with significant EBV viral reactivation (above 300 000 cp/mL, 30 000 IU/mL) as well as CMV reactivations (Table 5, Figures S2 and S3).

**TABLE 5:** Progression‐free survival for patients transplanted 2013 onwards.

	Unless otherwise specified	All patients			Type of MS								
					Secondary progressive				Primary progressive		Relapsing remitting		
		*N*	% 3 year PFS	95% CI	*N*	% 3 year PFS	95% CI	*N*	% 3 year PFS	95% CI	*N*	% 3 year PFS	95% CI
PFS from D90	Total	259	75%	68–80%	59	70%	55–82%	39	61%	42–76%	161	80%	72–86%
PFS from D90 by Baseline EDSS	≤3.0 (reference)	35	89%	68–96%	0	‐	‐	3	33%	1–77%	32	96%	73–99%
	3.5–5.5	78	69%	55–79%	11	30%	4–62%	14	65%	31–85%	53	77%	61–87%
			*p* = 0.307	HR = 1.61		Reference			*p* = 0.113	HR = 0.31		*p* = 0.288	HR = 2.02
	≥6.0	146	75%	66–81%	48	79%	61–86%	22	64%	39–81%	76	75%	63–84%
			*p* = 0.196	HR = 1.75		*p* = 0.040	HR = 0.32		*p* = 0.075	HR = 0.29		*p* = 0.067	HR = 3.07
	Continuous EDSS	262	*p* = 0.481	HR = 1.06		*p* = 0.006	HR = 0.32		*p* = 0.498	HR = 0.88		*p* = 0.286	HR = 1.12
PFS from D90 by ATG dose	≤6.0 mg/kg	103	84%	75–90%	15	64%	30–85%	6	60%	13–88%	83	88%	78–94%
	≥7.5 mg/kg	156	68%	58–75%	45	72%	54–84%	33	61%	40–76%	78	68%	54–79%
			*p* = 0.0005	HR = 2.52		*p* = 0.916	HR = 1.06		*p* = 0.621	HR = 0.73		*p* = 0.001	HR = 3.31
EBV reactivation	Non‐significant or none	209	81%		45	76%		28	72%		136	84%	
	Significant EBV	50	19%		14	24%		11	28%		25	16%	
PFS from D90 by EBV reactivation	Non‐significant or none	209	77%	70–83%	45	66%	48–79%	28	55%	32–73%	136	85%	77–91%
	Significant EBV	50	65%	49–77%	14	83%	45–95%	11	72%	35–90%	25	51%	29–70%
			*p* = 0.035	HR = 1.75		*p* = 0.501	HR = 0.68		*p* = 0.53	HR = 0.69		*p* = 0.0005	HR = 3.68
PFS from D90 by EBV and ATG dose	Non‐significant EBV & ATG ≤6.0	99	85%	76–91%	14	64%	30–85%	6	60%	13–88%	79	90%	81–85%
			Reference										
	Non‐significant EBV & ATG ≥ 7.5	110	67%	55–77%	31	67%	44–82%	22	52%	26–73%	57	75%	57–87%
			*p* = 0.001	HR = 2.60		*p* = 0.720	HR = 1.24		*p* = 0.216	HR = 0.82		*p* = 0.010	HR = 2.87
	Significant EBV & ATG ≤ 6.0	4	50%	6–84%	0	‐	‐	0	‐	‐	4	50%	6–84%
			*p* = 0.139	HR = 3.01								*p* = 0.066	HR = 4.10
	Significant EBV & ATG ≥ 7.5	46	66%	50–79%	14	83%	45–95%	11	72%	35–90%	21	52%	28–72%
			*p* = 0.001	HR = 2.88		*p* = 0.739	HR = 0.79		*p* = 0.498	HR = 0.60		*p* = 0.0005	HR = 6.15
CMV reactivation	None or < 1000 and untreated	223	86%		52	88%		27	69%		144	89%	
	>1000 or treated	36	14%		7	12%		12	31%		17	11%	
PFS by CMV reactivation	None or < 1000 and untreated	223	76%	69–82%	52	70%	54–82%	27	62%	37–79%	144	81%	73–87%
	>1000 or treated	36	66%	46–79%	7	75%	13–96%	12	49%	19–73%	17	35%	2–76%
			*p* = 0.042	HR = 1.81		*p* = 0.928	HR = 0.93		*p* = 0.976	HR = 0.98		*p* = 0.023	HR = 2.61
PFS by CMV reactivation and ATG dose	No CMV or <1000 ATG ≤ 6.0	100	83%	74–90%	14	64%	30–85%	6	60%	13–88%	80	88%	78–93%
			Reference										
	No CMV or < 1000 ATG ≥ 7.5	123	69%	58–78%	38	72%	52–84%	21	60%	31–80%	64	70%	53–82%
			*p* = 0.005	HR = 2.21		*p* = 0.899	HR = 1.08		*p* = 0.608	HR = 0.70		*p* = 0.009	HR = 2.65
	CMV > 1000 or treated ATG ≤ 6.0	3	100%	‐	0			0			3	100	‐
	CMV > 1000 or treated ATG ≥ 7.5	33	62%	41–77%	7	75%	13–96%	12	58%	27–80%	14	62%	31–82%
			*p* = 0.001	HR = 3.20		*p* = 0.987	HR = 0.99		*p* = 0.699	HR = 0.76		*p* = 0.0005	HR = 5.77

Abbreviations: AHSCT, autologous haematopoietic stem cell transplant; ATG, anti‐thymocyte globulin; CMV, cytomegalovirus; D, days; EBV, Epstein–Barr virus; EDSS, Expanded Disability Status Scale; HR, hazard ratio; MS, multiple sclerosis; *N*, number; PFS, progression‐free survival.

## DISCUSSION

This is the largest report on AHSCT for pwMS from the United Kingdom, where activity has steadily increased apart from a temporary drop‐off in activity in 2021–2022 due to the COVID‐19 pandemic. Most activity occurred post‐2016 in line with the increasing evidence base and funding support through 2013 NHSE Commissioning Policy.^25^ The United Kingdom has been a leader in Europe for AHSCT for pwMS, yet the UK map shows activity concentrated around two major metropolitan centres, London and Sheffield, with large areas reporting little AHSCT activity for MS. There is a referral pathway for patients from the Republic of Ireland; however, the rest of the UK‐devolved nations, in particular Scotland and Northern Ireland, have shown no activity. NHS Wales commissioners have recently agreed a pathway, but this still means patients travelling to Sheffield. There is a need for the provision of expertise across the United Kingdom to drive equitable geographical access and meet increasing patient demands, supported by health economic analysis.^31^ The STAR‐MS study,^32^ which recently completed recruitment at 13 UK centres, may help through establishing multidisciplinary networks of support.^33^

Our UK cohort is distinct in including 36% progressive MS patients historically excluded from randomised studies of AHSCT. Many patients had advanced disability at time of AHSCT (median EDSS 6.0) along with median failure of at least one high efficacy DMT. Prognosis for this ‘difficult to treat’ MS group is poor with rapid progression of disability. In this ‘real‐world’ cohort, 62% of patients remain free of progression at 5 years post‐AHSCT. Higher disability at time of AHSCT and primary progressive MS were associated with worse PFS.

This paper identifies specific issues during AHSCT for MS, including poor tolerability of conditioning in those with advanced MS related to impaired cardio‐respiratory reserve not picked up on routine transplant work‐up tests. Other factors to be considered in patients with MS are the increased risk of seizures, poor mobility and falls risks, poor tolerance of rATG fever and incomplete bladder voiding. Overall TRM by day +100 was 1.4% with all patients affected having advanced levels of disability. TRM occurred very early in the transplant course, mainly starting during conditioning before the aplastic phase and potentially related to reduced cardio‐respiratory reserve in patients with advanced MS compounded by conditioning regimens (such as cyclophosphamide‐ATG) that induce significant fluid retention and direct cardiotoxicity which may result in rapid clinical deterioration. It is vital to judiciously monitor fluid balance (once/twice a day), exercise careful diuretic therapy and pay close attention to electrolyte levels, alongside careful management of fever. In addition to traditional infection management, consideration should be given to administration of high doses of methylprednisolone (250–500 mg iv given stat) for any fever persisting beyond 1 h. MS patients are also at higher risk of seizures and maintaining good electrolyte levels is vital. Mobility and bladder infections highlight the need to consider pre‐emptive catheterisation for patients with incomplete bladder emptying to reduce infection risk and facilitate fluid management.^34^ Falls risk, particularly during the thrombocytopenic phase of AHSCT, requires a multidisciplinary approach involving the nursing team, physiotherapists and occupational therapists, with careful assessment of patients baseline functional status and support to aid recovery following AHSCT. Patients should be treated within rooms which make allowance for reduced mobility, poor balance and deconditioning due to treatment.^34^, ^35^

Viral reactivation, particularly EBV, remains a significant issue and routine monitoring is mandatory for the first 100 days. EBV is intrinsically linked with the development of MS, as reflected by the high seroprevalence of EBV in the MS population compared with the general population (Table 1). We previously reported that uncontrolled EBV reactivation resulted in clinically significant sequelae for patients.^30^ This study reveals an interaction of reduced PFS and increased morbidity associated with an increased risk of viral reactivations, fluid overload, rATG dose and advanced EDSS (Table 5). Based on this, the Pan‐London group recently adjusted the EDSS threshold for ASHCT eligibility to 6.0 or less. They also recommended a reduction in the rATG dose to 6.0 mg/kg total to mitigate against the increased risk of viral reactivations (Table 3). The previous EBV reactivation treatment threshold of 500 000 cp/mL (50 000 IU/mL) has also been reduced by the Pan‐London group in light of the findings in this paper of adverse outcomes with reactivation above 300 000 cp/mL (30 000 IU/mL). A recent survey of transplant centres across Europe showed that 36.9% of centres used doses of 7.5 mg/kg or more of r‐ATG and it remained unclear if higher ATG doses conferred better disease control.^36^ This report, for the first time, confirms no benefit and potential risks of higher ATG doses (>6.0 mg/kg). Therefore, it seems reasonable to cap the rATG dose to 6.0 mg/kg.

Our report highlights the importance of knowledge and learning exchange. Recognition of MS specific issues during AHSCT led to the harmonisation of MS‐specific AHSCT protocols across the United Kingdom alongside the development of a Pan‐London MDT. The UK experience has contributed to the recent recommendations for AHSCT in MS and related disorders produced by ECTRIMS and EBMT.^34^, ^37^

Study limitations include the retrospective study design and lack of comparator DMT arm preventing direct comparative safety and effectiveness analysis. Understanding the intensity of the immune ablation is also vital as not all transplants are of equal intensity. The recently published paper from the team in Mexico highlighted ‘the Mexican Method’ for AHSCT in 1700 patients, but the follow‐up was more restricted than in our series where more robust neurological follow‐up was performed. Comparison between the two protocols is therefore not possible in terms of safety and outcome.^38^

In conclusion, AHSCT remains a very effective one‐off therapy for treatment of patients with severe MS. Careful patient selection by a multidisciplinary team is important to optimise risk/benefit and data collection is vital for meaningful ‘real‐world’ outcome analysis. Ongoing randomised prospective trials comparing efficacy of AHSCT versus high‐efficacy disease‐modifying therapy in RRMS, including STAR‐MS^33^ in the United Kingdom, expand the experience and capacity and will provide strong evidence on the position of AHSCT in the treatment for pwMS.

## CONFLICT OF INTEREST STATEMENT

Paolo A. Muraro has received fees from consulting to Cellerys, unrelated to this study; John A. Snowden declares advisory boards for Vertex, Jazz, Medac and BMS, not related to this study.

## Supporting information


Figure S1.

Figure S2.

Figure S3.

## Data Availability

Anonymised data not published within this article will be made available by request from any qualified investigator.

## References

[1] Jakimovski D , Bittner S , Zivadinov R , Morrow SA , Benedict RH , Zipp F , et al. Multiple sclerosis. Lancet. 2024;403(10422):183–202. 37949093 10.1016/S0140-6736(23)01473-3

[2] Lunde HMB , Assmus J , Myhr K‐M , Bø L , Grytten N . Survival and cause of death in multiple sclerosis: a 60‐year longitudinal population study. J Neurol Neurosurg Psychiatry. 2017;88(8):621–625. 28365589 10.1136/jnnp-2016-315238 PMC5537547

[3] MS Society . MS in the UK. https://www.mssociety.org.uk/what‐we‐do/our‐work/our‐evidence/ms‐in‐the‐uk. MS Society 2024. Available from: https://www.mssociety.org.uk/what‐we‐do/our‐work/our‐evidence/ms‐in‐the‐uk

[4] Alonso A , Hernán MA . Temporal trends in the incidence of multiple sclerosis: a systematic review. Neurology. 2008;71(2):129–135. 18606967 10.1212/01.wnl.0000316802.35974.34 PMC4109189

[5] Kister I , Bacon TE , Chamot E , Salter AR , Cutter GR , Kalina JT , et al. Natural history of multiple sclerosis symptoms. Int J MS Care. 2013;15(3):146–158. 24453777 10.7224/1537-2073.2012-053 PMC3883021

[6] Thompson AJ , Banwell BL , Barkhof F , Carroll WM , Coetzee T , Comi G , et al. Diagnosis of multiple sclerosis: 2017 revisions of the McDonald criteria. Lancet Neurol. 2018;17(2):162–173. 29275977 10.1016/S1474-4422(17)30470-2

[7] Goodin DS . The epidemiology of multiple sclerosis: insights to disease pathogenesis. Handb Clin Neurol. 2014;122:231–266. 24507521 10.1016/B978-0-444-52001-2.00010-8

[8] Sabatino JJ , Pröbstel A‐K , Zamvil SS . B cells in autoimmune and neurodegenerative central nervous system diseases. Nat Rev Neurosci. 2019;20(12):728–745. 31712781 10.1038/s41583-019-0233-2

[9] Dendrou CA , Fugger L , Friese MA . Immunopathology of multiple sclerosis. Nat Rev Immunol. 2015;15(9):545–558. 26250739 10.1038/nri3871

[10] Bjornevik K , Cortese M , Healy BC , Kuhle J , Mina MJ , Leng Y , et al. Longitudinal analysis reveals high prevalence of Epstein‐Barr virus associated with multiple sclerosis. Science. 2022;375(6578):296–301. 35025605 10.1126/science.abj8222

[11] Cohen JA , Coles AJ , Arnold DL , Confavreux C , Fox EJ , Hartung HP , et al. Alemtuzumab versus interferon beta 1a as first‐line treatment for patients with relapsing‐remitting multiple sclerosis: a randomised controlled phase 3 trial. Lancet. 2012;380(9856):1819–1828. 23122652 10.1016/S0140-6736(12)61769-3

[12] Hauser SL , Bar‐Or A , Comi G , Giovannoni G , Hartung H‐P , Hemmer B , et al. Ocrelizumab versus interferon Beta‐1a in relapsing multiple sclerosis. N Engl J Med. 2017;376(3):221–234. 28002679 10.1056/NEJMoa1601277

[13] Hauser SL , Bar‐Or A , Cohen JA , Comi G , Correale J , Coyle PK , et al. Ofatumumab versus Teriflunomide in Multiple Sclerosis. N Engl J Med. 2020;383(6):546–557. 32757523 10.1056/NEJMoa1917246

[14] Polman CH , O'Connor PW , Havrdova E , Hutchinson M , Kappos L , Miller DH , et al. A randomized, placebo‐controlled trial of Natalizumab for relapsing multiple sclerosis. N Engl J Med. 2006;354(9):899–910. 16510744 10.1056/NEJMoa044397

[15] Montalban PX , editor. Update of the ECTRIMS/EAN guidelines on the treatment of multiple sclerosis. Baarn, Netherlands: Medicom Medical Publishers; 2021.

[16] Kappos L , Wolinsky JS , Giovannoni G , Arnold DL , Wang Q , Bernasconi C , et al. Contribution of relapse‐independent progression vs relapse‐associated worsening to overall confirmed disability accumulation in typical relapsing multiple sclerosis in a pooled analysis of 2 randomized clinical trials. JAMA Neurol. 2020;77(9):1132–1140. 32511687 10.1001/jamaneurol.2020.1568 PMC7281382

[17] Coles AJ , Cohen JA , Fox EJ , Giovannoni G , Hartung HP , Havrdova E , et al. Alemtuzumab CARE‐MS II 5‐year follow‐up: efficacy and safety findings. Neurology. 2017;89(11):1117–1126. 28835403 10.1212/WNL.0000000000004354 PMC5595276

[18] Muraro PA , Martin R , Mancardi GL , Nicholas R , Sormani MP , Saccardi R . Autologous haematopoietic stem cell transplantation for treatment of multiple sclerosis. Nat Rev Neurol. 2017;13(7):391–405. 28621766 10.1038/nrneurol.2017.81

[19] Muraro PA . Resetting tolerance in autoimmune disease. Science. 2023;380(6644):470–471. 37141350 10.1126/science.adg7489

[20] Alexander T , Greco R . Hematopoietic stem cell transplantation and cellular therapies for autoimmune diseases: overview and future considerations from the autoimmune diseases working party (ADWP) of the European Society for Blood and Marrow Transplantation (EBMT). Bone Marrow Transplant. 2022;57(7):1055–1062. 35578014 10.1038/s41409-022-01702-w PMC9109750

[21] Sormani MP , Muraro PA , Schiavetti I , Signori A , Laroni A , Saccardi R , et al. Autologous hematopoietic stem cell transplantation in multiple sclerosis: a meta‐analysis. Neurology. 2017;88(22):2115–2122. 28455383 10.1212/WNL.0000000000003987

[22] Mancardi GL , Sormani MP , Gualandi F , Saiz A , Carreras E , Merelli E , et al. Autologous hematopoietic stem cell transplantation in multiple sclerosis: a phase II trial. Neurology. 2015;84(10):981–988. 25672923 10.1212/WNL.0000000000001329

[23] Burt RK , Balabanov R , Burman J , Sharrack B , Snowden JA , Oliveira MC , et al. Effect of Nonmyeloablative hematopoietic stem cell transplantation vs continued disease‐modifying therapy on disease progression in patients with relapsing‐remitting multiple sclerosis: a randomized clinical trial. JAMA. 2019;321(2):165–174. 30644983 10.1001/jama.2018.18743 PMC6439765

[24] Boffa G , Massacesi L , Inglese M , Mariottini A , Capobianco M , Lucia M , et al. Long‐term clinical outcomes of hematopoietic stem cell transplantation in multiple sclerosis. Neurology. 2021;96(8):e1215–e1226. 33472915 10.1212/WNL.0000000000011461

[25] Dignan F , Potter V , Davies E , Griffin J , Slatter M , Bloor A , et al. NHS commissioning—blood marrow transplantation 2013. Available from: https://www.england.nhs.uk/commissioning/spec‐services/npc‐crg/blood‐and‐infection‐group‐f/blood‐and‐marrow‐transplantation/

[26] Robles‐Nasta M , Lira‐Lara O , Olivares‐Gazca JC , Gomez‐Almaguer D , Gomez‐de Leon A , Ruiz‐Delgado GJ , et al. Why are persons with multiple sclerosis traveling to Mexico to obtain a hematopoietic stem cell transplant? Med Univ. 2024;26(4):105–108.

[27] Nash RA , Hutton GJ , Racke MK , Popat U , Devine SM , Steinmiller KC , et al. High‐dose immunosuppressive therapy and autologous HCT for relapsing‐remitting MS. Neurology. 2017;88(9):842–852. 28148635 10.1212/WNL.0000000000003660 PMC5331868

[28] Atkins HL , Bowman M , Allan D , Anstee G , Arnold DL , Bar‐Or A , et al. Immunoablation and autologous haemopoietic stem‐cell transplantation for aggressive multiple sclerosis: a multicentre single‐group phase 2 trial. Lancet. 2016;388(10044):576–585. 27291994 10.1016/S0140-6736(16)30169-6

[29] Muraro P , Kazmi M , De Matteis E , Brittain G , Mariottini A , Nicholas R , et al. Real‐world effectiveness of autologous haematopoietic stem cell transplantation for MS in the UK submitted. 2025. 10.1136/jnnp-2025-336755 40912910

[30] Mehra V , Rhone E , Widya S , Zuckerman M , Potter V , Raj K , et al. Epstein‐Barr virus and monoclonal Gammopathy of clinical significance in autologous stem cell transplantation for multiple sclerosis. Clin Infect Dis. 2019;69(10):1757–1763. 30980715 10.1093/cid/ciz047

[31] Hughes SL , Prettyjohns MJ , Snowden JA , Sharrack B . Economics of hematopoietic stem cell transplant in immune‐mediated neurologic autoimmune diseases. Handb Clin Neurol. 2024;202:279–294. 39111914 10.1016/B978-0-323-90242-7.00007-9

[32] Autologous stem cell transplantation versus alemtuzumab, ocrelizumab, ofatumumab or cladribine in relapsing‐remitting multiple sclerosis. 2020. Available from: https://www.isrctn.com/ISRCTN88667898 10.1136/bmjopen-2023-083582 PMC10860024 38316583

[33] Brittain G , Petrie J , Duffy KEM , Glover R , Hullock K , Papaioannou D , et al. Efficacy and safety of autologous haematopoietic stem cell transplantation versus alemtuzumab, ocrelizumab, ofatumumab or cladribine in relapsing remitting multiple sclerosis (StarMS): protocol for a randomised controlled trial. BMJ Open. 2024;14(2):e083582. 10.1136/bmjopen-2023-083582 PMC10860024 38316583

[34] Ismail A , Sharrack B , Saccardi R , Moore JJ , Snowden JA . Autologous haematopoietic stem cell therapy for multiple sclerosis: a review for supportive care clinicians on behalf of the autoimmune diseases working Party of the European Society for blood and marrow transplantation. Curr Opin Support Palliat Care. 2019;13(4):394–401. 31599815 10.1097/SPC.0000000000000466 PMC6867671

[35] Roberts F , Hobbs H , Jessop H , Bozzolini C , Burman J , Greco R , et al. Rehabilitation before and after autologous Haematopoietic stem cell transplantation (AHSCT) for patients with multiple sclerosis (MS): consensus guidelines and recommendations for best clinical practice on behalf of the autoimmune diseases working party, nurses group, and patient advocacy Committee of the European Society for blood and marrow transplantation (EBMT). Front Neurol. 2020;11:556141. 33362684 10.3389/fneur.2020.556141 PMC7759663

[36] Ismail A , Nitti R , Sharrack B , Badoglio M , Ambron P , Labopin M , et al. ATG and other serotherapy in conditioning regimens for autologous HSCT in autoimmune diseases: a survey on behalf of the EBMT autoimmune diseases working party (ADWP). Bone Marrow Transplant. 2024;59(11):1614–1617. 39143182 10.1038/s41409-024-02383-3 PMC11530368

[37] Muraro PA , Mariottini A , Greco R , Burman J , Iacobaeus E , Inglese M , et al. Autologous haematopoietic stem cell transplantation for treatment of multiple sclerosis and neuromyelitis optica spectrum disorder—recommendations from ECTRIMS and the EBMT. Nat Rev Neurol. 2025;21:140–158. 39814869 10.1038/s41582-024-01050-x

[38] Lira‐Lara O , Robles‐Nasta M , Olivares‐Gazca JC , Kharfan‐Dabaja M , Rivera‐Álvarez M , García‐Vélez D , et al. Early morbimortality in autologous hematopoietic cell transplantation performed on outpatient basis in patients with autoimmune diseases: experience in 1700 patients. Bone Marrow Transplant. 2025;60:640–644. 40069376 10.1038/s41409-025-02544-y

