# Supplementary figures and images for: Autologous haematopoietic stem cell transplantation for multiple sclerosis in the UK: A 20‐year retrospective analysis of activity and haematological outcomes from the British Society of Blood and Marrow Transplantation and Cellular Therapy (BSBMTCT)
Source: Br J Haematol. 2025 Jun 11;207(3):977–91. doi: 10.1111/bjh.20199 (PMC12436235; doi:10.1111/bjh.20199)

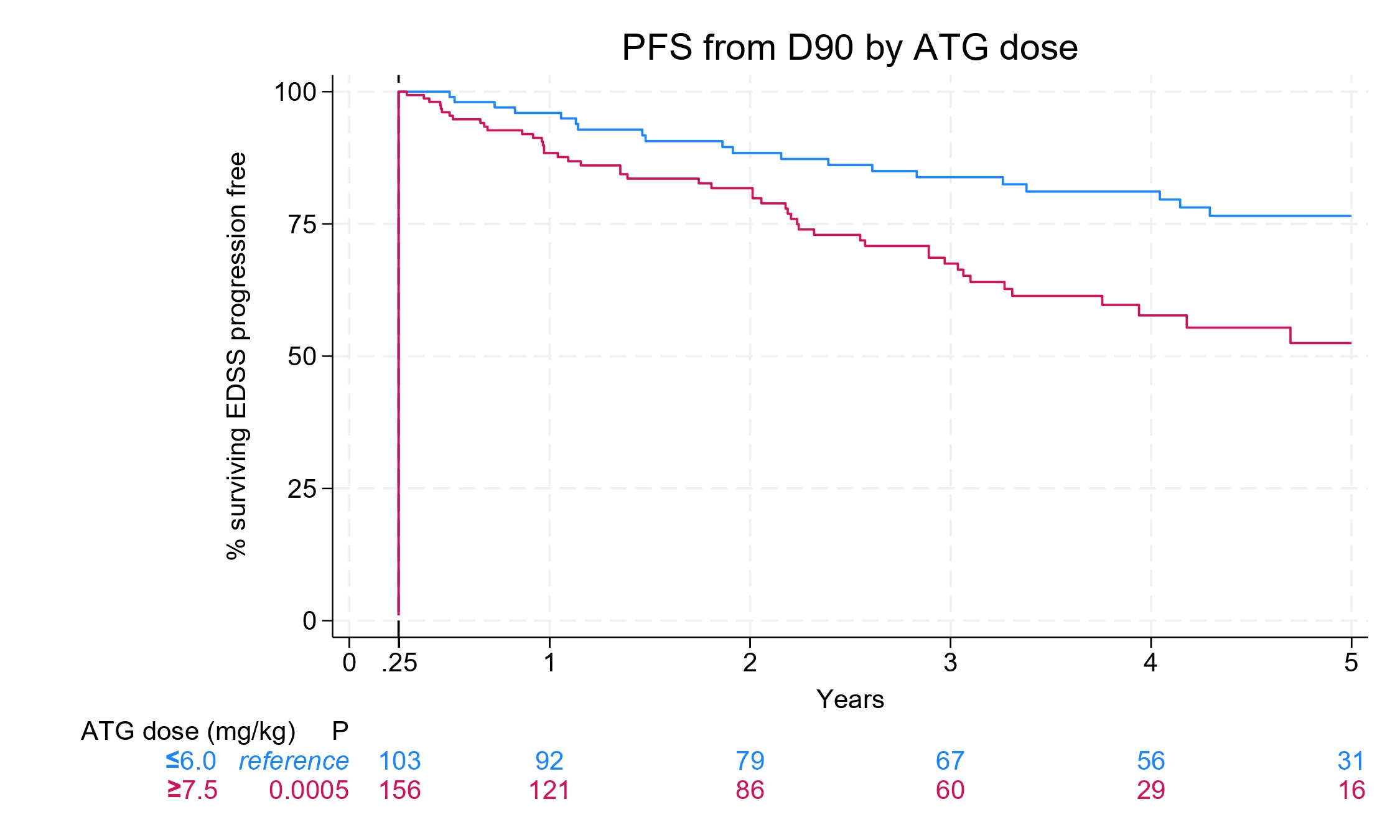

Supplement: Supplementary file 1 — Figure S1. [file BJH-207-977-s001.jpg]

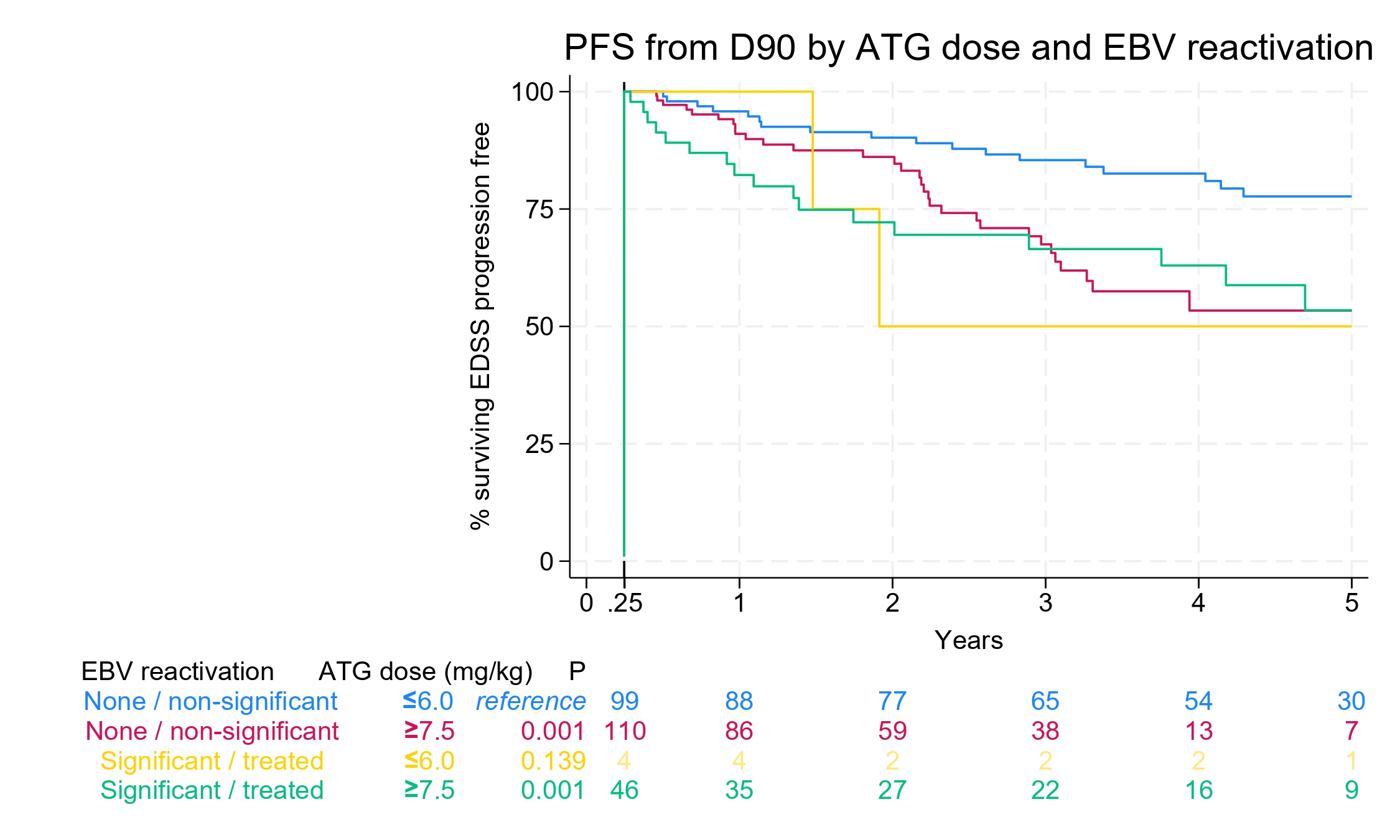

Supplement: Supplementary file 2 — Figure S2. [file BJH-207-977-s002.jpg]

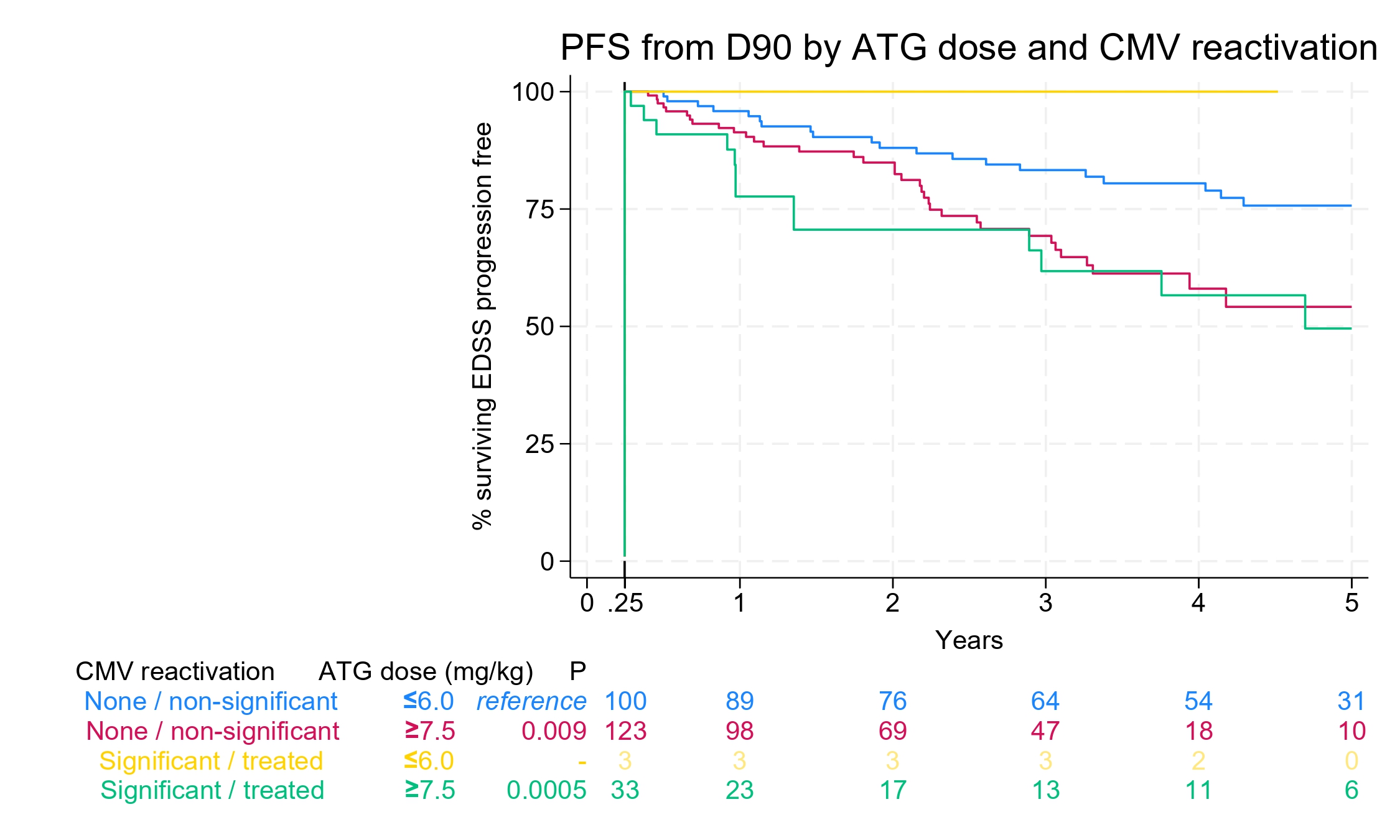

Supplement: Supplementary file 3 — Figure S3. [file BJH-207-977-s003.jpg]
